# Blocking salt‐inducible kinases with YKL‐06‐061 prevents PTZ‐induced seizures in mice

**DOI:** 10.1002/brb3.3305

**Published:** 2023-11-02

**Authors:** Lixuan Peng, Cai Li, Xiaohan Tang, Yuyan Xiang, Yang Xu, Wenyu Cao, Huamao Zhou, Suyun Li

**Affiliations:** ^1^ Clinical Anatomy and Reproductive Medicine Application Institute, Hengyang Medical School University of South China Hengyang Hunan China; ^2^ Institute of Neuroscience, Hengyang Medical School University of South China Hengyang Hunan China; ^3^ Nanhua Affiliated Hospital, Hengyang Medical School University of South China Hengyang Hunan China

**Keywords:** epilepsy, neuronal activation, salt‐inducible kinases, YKL‐06‐061

## Abstract

**Introduction:**

Epilepsy is one of the most common neurological diseases, while over one third of adults with epilepsy still have inadequate seizure control. Although mutations in salt‐inducible kinases (SIKs) have been identified in epileptic encephalopathy, it is not known whether blocking SIKs can prevent pentylenetetrazole (PTZ)‐induced seizures.

**Methods:**

We first determined the time course of SIKs (including SIK 1, 2, and 3) in the hippocampus of PTZ treated mice. And then, we evaluated the effects of anti‐epilepsy drug valproate acid (VPA) on the expression of SIK 1, 2, and 3 in the hippocampus of PTZ treated mice. Next, we investigated the effect of different dose of SIKs inhibitor YKL‐06‐061 on the epileptic seizures and neuronal activation by determining the expression of immediate early genes (IEGs) in the PTZ treated mice.

**Results:**

We found that PTZ selectively induced enhanced expression of SIK1 in the hippocampus, which was blocked by VPA treatment. Notably, YKL‐06‐061 decreased seizure activity and prevented neuronal overactivity, as indicated by the reduced expression of IEGs in the hippocampus and prefrontal cortex.

**Conclusion:**

Our findings provide the first evidence that SIK1 affects gene regulation in neuronal hyperactivity, which is involved in seizure behavior. Targeting SIK1 through the development of selective inhibitors may lead to disease‐modifying therapies that reduce epilepsy progression.

## Graphical abstract

A schematic illustration of the proposed mechanism for YKL‐06‐061 prevents PTZ‐induced seizures in mice. PTZ induces high expression of SIK1, leading IEGs overexpression and appearing seizure behavior in mice. SIKs inhibitors (YKL‐06‐061) reduce the expression of IEGs, thereby improving epilepsy of mice.

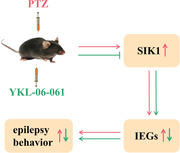

## 1 INTRODUCTION

Epilepsy, a disease with a prevalence of approximately 1%, is one of the most common neurological diseases affecting approximately 50 million people worldwide (Fiest et al., 2017). Although more than a dozen new anti‐seizure medications have been used in the last two decades, over one third of adults with epilepsy still have inadequate seizure control (Tipton et al., 2023). Unfortunately, recurrent or prolonged seizures may lead to permanently altered neuronal circuitry, excitotoxic injury, and aberrant inflammation (Eyo et al., 2017). As aberrant neuronal activity induces pathological network changes that contribute to the subsequent progression of the seizure burden, neuronal over‐excitation is considered an essential feature across different types of epileptic seizure models (Knowles et al., 2022). Thus, an improved understanding of the mechanisms underlying seizure progression, particularly neuronal overactivation, could facilitate the development of innovative anti‐epileptogenic treatment strategies.

Salt‐inducible kinases (SIKs), including SIK1, SIK2, and SIK3, belong to the AMP‐activated protein kinase (AMPK) family, which mainly regulates energy response‐related physiological processes, such as gluconeogenesis and lipid metabolism (Badawi et al., 2021). In addition, SIK family members are dysregulated in various cancers, including ovarian, breast, prostate, and lung cancers (Sun et al., 2020). Their functions in the central nervous system are better understood. SIK1 is abundantly expressed in the adrenal cortex, as well as in adipose and neural tissues, whereas both SIK2 and SIK3 are ubiquitous in humans and are mainly expressed in adipose and neural tissues, respectively (Jagannath et al., 2013). In particular, SIK1 and SIK2 are associated with neuropsychiatric disorders, and the inhibition of SIKs has been shown to be effective in treating these neuropsychiatric disorders (Hansen et al., 2015; Liu et al., 2020). Notably, SIK mRNA levels were significantly increased in the rat hippocampus and cortex following kainic acid (KA)‐induced seizures (Feldman et al., 2000). Moreover, individuals with SIK1 mutations have shorter survival in cases of neonatal epilepsy onset and autism plus developmental syndrome after infantile spasms in others (Hansen et al., 2015). Furthermore, SIK1 mutant mice, recapitulating the C‐terminal‐truncated mutation of SIK1, displayed enhanced seizure susceptibility (Pang et al., 2022). Despite recent reports revealing the role of SIKs in the development of epileptic seizures (Proschel et al., 2017), the causal link and mechanisms underlying the participation of SIKs in epileptogenesis remain poorly understood. Moreover, whether SIK inhibitors could be effective targets for epilepsy treatment needs to be investigated.

In this study, to investigate the contribution of SIKs in regulating seizure susceptibility in vivo, we used a mouse model of acute seizures chemically induced by administering the potent and noncompetitive gamma‐aminobutyric acid type A receptor antagonist pentylenetetrazole (PTZ) (Hosseini‐Zare et al., 2011). Having elucidated the time course of SIKs in seizure progression, we explored whether the expression of SIKs in PTZ‐treated mice was altered by valproic acid (VPA), an anticonvulsant agent (Khan et al., 2020). We further investigated whether YKL‐06‐061, a selective second‐generation inhibitor of SIKs (Mujahid et al., 2017), could prevent PTZ‐induced seizures. We next examined the effect of YKL‐06‐061 on neuronal hyperactivity as indicated by the identification of immediate early genes (IEGs) in the brain. To the best of our knowledge, this is the first comprehensive study to show that YKL‐06‐061 has beneficial effects against seizures.

## 2 EXPERIMENTAL PROCEDURES

### 2.1 Experimental animals

Male C57BL/6J mice were used for the present experiments, which were purchased from the Hunan SJA Lab Animal Center of Changsha, Hunan, China. The animals weighed 22–25 g and were 8‐week old. Mice were group‐housed under controlled conditions (12 h light/dark cycle, 20–25°C, and 40%–60% relative humidity) with free access to food and water. Before establishing the model, the mice were allowed to adapt to their new environment for 7 days. The experimental protocol was approved by the Animal Care and Use Committee of the University of South China (permit number: USC2020031602) and was conducted in accordance with the National Institutes of Health Guide for the Care and Use of Laboratory Animals.

The expression of SIK1, 2, 3 was evaluated in the hippocampus using RT‐PCR analysis at 1, 1.5, and 2 h after PTZ injection and compared to that in normal saline (NS)‐treated animals. The spatial expression of SIK1 was further investigated using immunohistochemistry assay.

To establish the relationship between SIKs and seizure behavior, the animals were intraperitoneally (i.p.) administered VPA or NS and i.p. injected with PTZ 30 min later. The expression of SIK1, 2, and 3 was evaluated in the hippocampus using RT‐PCR analysis 1 h after PTZ injection.

To further explore the causal association between SIKs and seizure behavior, the animals were first i.p. treated with the SIK inhibitor YKL‐06‐061 or vehicle and were then i.p. injected with PTZ 30 min later. The expression of IEGs in the hippocampus was evaluated using RT‐PCR or immunohistochemistry assay at 1 h after PTZ administration.

### 2.2 Drugs

To induce epilepsy in mice, we used the PTZ (P6500, SIGMA) induction method. Male C57BL/6J mice were treated with a single i.p. injection of PTZ (45 mg/kg) (dissolved in NS) to establish an animal model of epilepsy, whereas control mice were treated with the same amount of NS. In SIK inhibitor treatment experiments, YKL‐06‐061 (dissolved in dimethyl sulfoxide [DMSO]) or the same amount of vehicle was injected for 7 days. Here, the dose of YKL‐06‐061 was administered in a concentration gradient with 10, 20, or 40 mg/kg to screen for the optimal dose. The mice were randomly divided into DMSO + NS, DMSO + PTZ, YKL + NS, and YKL + PTZ groups. Thirty minutes after the last treatment with YKL or vehicle, the animals were i.p. injected with PTZ or NS, and their seizure behavior was observed for 1 h.

### 2.3 Epilepsy score

Seizure behavior was determined according to the modified Racine scale: the normal activity was defined as level 0; vertical tail and body shaking meter was level 1; local clonic seizure (only bilateral forelimb or unilateral limb twitching) was classified as level 2; systemic clonic seizure (limb twitching) was level 3; ankylotic convulsion (forelimb flexion, hind limb stiffness, or rollover) was level 4; systemic tetanic convulsion (TC) and death count were defined as level 5 (Van Erum et al., 2019). The time of part clonic (PC), general clonic (GC), and TC, and the seizure level of mice were recorded and analyzed. If no acute seizures were observed in mice and the mice only had symptoms such as erect tail and body shaking, the latency of the local clonic seizure was recorded as 30 min, and the seizure level was recorded as level 1. The susceptibility scores of mice = (0.2) (1/PC latency) + (0.3) (1/GC latency) + (0.5) (1/TC latency) (Korb et al., 2015). The higher the epilepsy susceptibility score, the more prone the mice were to epilepsy.

### 2.4 Immunohistochemistry

After completion of the behavioral test, the animals were sacrificed for immunohistochemistry assay, according to our previous study (Wang et al., 2020). After being anesthetized with 10% sodium pentobarbital, the mice were perfused with saline and 4% paraformaldehyde. After perfusion, the brains were removed into 4% paraformaldehyde at 4°C overnight and were then dehydrated in 15% and 30% sucrose solution at 4°C. Cryostat was used to cut mice brains into 30 µm thick sections. To remove endogenous peroxidase, the sections were treated with 3% H_2_O_2_ for 25 min, and the free‐floating sections were then washed with 0.01 M phosphate buffered saline three times (10 min each time). The sections were blocked with 5% goat serum (with 0.1% Triton X‐100) for 2 h at room temperature and were incubated with the primary antibodies for 2 h at room temperature, including rabbit anti‐c‐fos primary antibody (1:1000, CST), rabbit anti‐Fos‐B primary antibody (1:1000, CST), rabbit anti‐Egr‐1 primary antibody (1:1000, CST), and rabbit anti‐SIK1 primary antibody (1:300, Affinity) before they were incubated at 4°C overnight. On the second day, the sections were washed and incubated with secondary reagents containing biotinylated goat anti‐rabbit immunoglobulin (Proteintech) for 2 h. The sections were treated with the peroxidase substrate diaminobenzidine tetrahydrochloride (ZSGB‐BIO). All images were captured using an optical microscope (Olympus) under identical conditions. Immunopositive c‐fos, Fos‐B, Egr‐1, and SIK1 cells were counted by a researcher blinded to the treatment.

### 2.5 Real time‐PCR

After being anesthetized with 4% sodium pentobarbital, the brains of mice were removed, and the hippocampus and prefrontal cortex were isolated and frozen at −80°C. The Trizol reagent (CWBIO) was used to extract total RNA according to the manufacturer's instructions. RNA purity was determined using the A260/A280 nm absorption ratio. A RevertAid First Strand cDNA Synthesis Kit (Fermentas) was used to synthesize cDNA according to the manufacturer's instructions. Gene expression levels were determined using an ABI‐7500 real‐time PCR system employing TB Green Premix Ex Taq II (Takara). The glyceraldehyde‐3‐phosphate dehydrogenase gene expression was used as an internal control. Primers were designed using the Primer 3 software (Table 1). The PCR cycling conditions were 30 s at 95°C followed by 40 cycles at 95°C for 5 s and 60°C for 45 s. The 2^−△△^ *^Ct^* method was used to determine the relative gene expression, according to our previous study (Lv et al., 2020).

**TABLE 1:** The primers used in this study.

Genes	Primers	Sequence 5′–3′
GAPDH	Forward	ACCACCATGGAGAAGGCTGG
GAPDH	Reverse	CTCAGTGTAGCCCAGGATGC
c‐fos	Forward	TCTCTAGTGCCAACTTTATCCC
c‐fos	Reverse	GAGATAGCTGCTCTACTTTGCC
Fos‐B	Forward	AGGCAGAGCTGGAGTCGGAGAT
Fos‐B	Reverse	GCCGAGGACTTGAACTTCACTCG
NPAS4	Forward	GCAGTCATGTACCGATCCACCAAG
NPAS4	Reverse	GCGAGTGTAGATGCAGGCAAGAC
Egr‐1	Forward	CCCAGGACTTAAAGGCTCTTAA
Egr‐1	Reverse	TGGTCACTACGACTGAAGTTAC
BDNF	Forward	CCCATGAAAGAAGTAAACGTC
BDNF	Reverse	CCTTATGGTTTTCTTCGTTGGG
SIK1	Forward	GACTACAACGAACAGGTGCTAG
SIK1	Reverse	GAGTAGGAGGTAGTAAATGGCG
SIK2	Forward	GCCTTGTGAAGACCGAGAACCATC
SIK2	Reverse	AGCCGTGGAACTAGGGATGTCTAC
SIK3	Forward	CTTGAACATGAATCGGTTCTCC
SIK3	Reverse	CTGTAGTGCTGACGTGGTATAG

Abbreviations: BDNF, brain‐derived neurotrophic factor; GAPDH, glyceraldehyde‐3‐phosphate dehydrogenase; NPAS4, neuronal PAS domain protein 4; SIK, salt‐inducible kinases.

### 2.6 Statistical analyses

Data analyses were performed using GraphPad Prism 8.0 software (GraphPad Software). Significant differences were determined using one‐way analysis of variance, followed by post hoc Bonferroni test for multiple comparisons between more than two groups. The values that were outside average ± 2.5 (standard deviations) were excluded as outliers (Kljakic et al., 2021; Ruigrok et al., 2021). The value of *p* < .05 was considered statistically significant.

## 3 RESULTS

### 3.1 SIK1 is selectively upregulated in the hippocampus in response to seizure activity induced by PTZ

**FIGURE 1:**
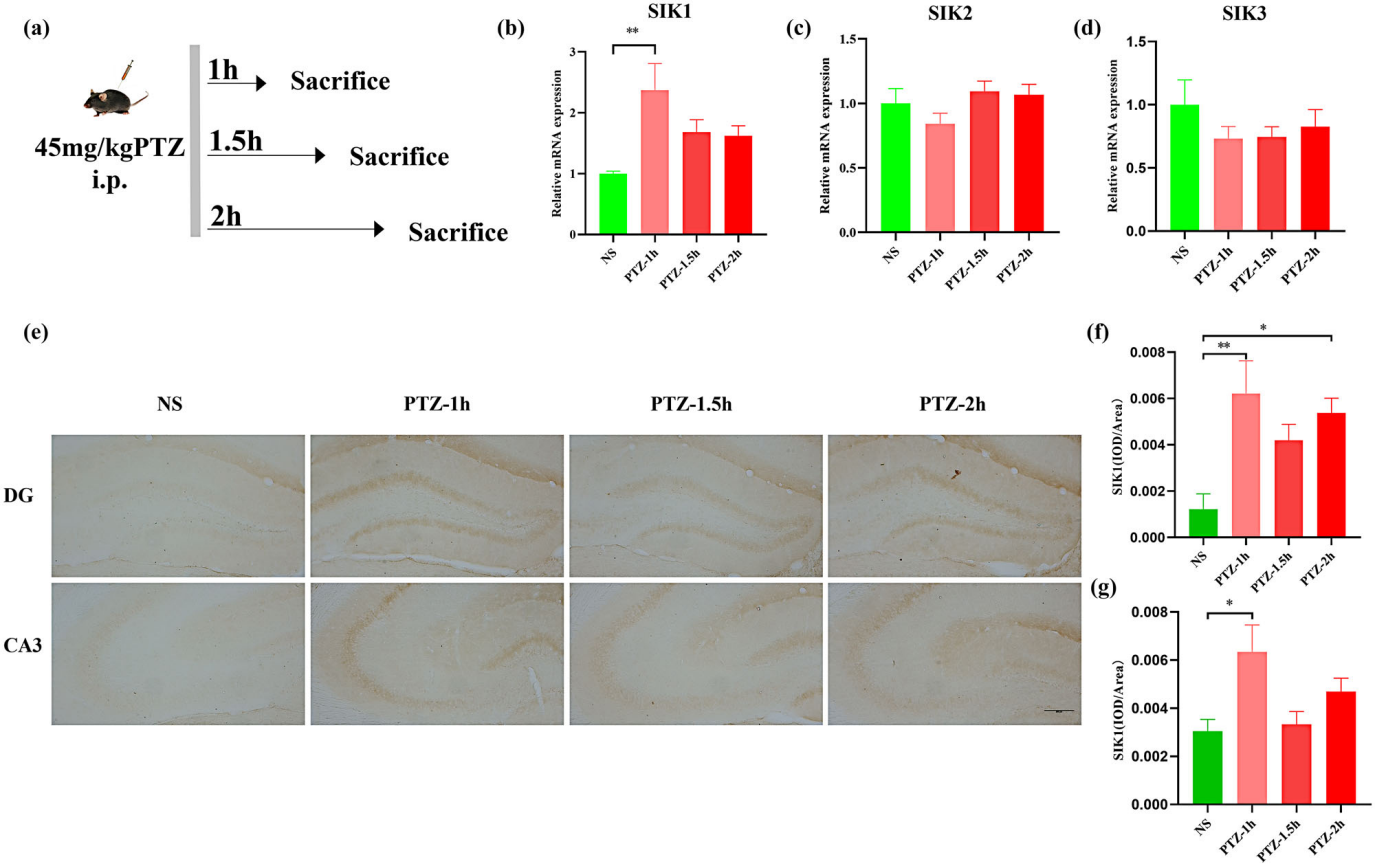
Expression of salt‐inducible kinases (SIKs) in normal mice and mice with pentylenetetrazole (PTZ)‐induced seizures in the hippocampus: (a) the timeline of PTZ molding; (b–d) representative mRNA expression levels of SIK1, SIK2, and SIK3, *N* = 6 per group; (e) representative SIK1 immunohistochemistry images in the dentate gyrus (DG) and cornu ammonis (CA3) (Bar = 200 µm); (f and g) quantification of the average optical density (OD) of SIK1 in the DG and CA3 regions of the hippocampus for each group, *N* = 3 per group. ^*^ *p* < .05, ^**^ *p* < .01, ^***^ *p* < .001, ^****^ *p* < .0001.

To explore alterations in SIKs in epilepsy, PTZ (45 mg/kg) was used to establish an in vivo epilepsy mouse model (Figure 1a). The expression of SIKs has been detected in the hippocampus, a brain region susceptible to epilepsy. RT‐PCR showed that when compared to the NS group, the mRNA levels of SIK1 (*t* = 3.780, *p* = .0071) in the PTZ‐1 h group increased significantly, leaving unaltered levels of SIK2 and SIK3 (Figure 1b–d). Furthermore, we used immunohistochemistry to investigate the expression of SIK1, and representative images of SIK1‐positive cells in the dentate gyrus (DG) and cornu ammonis (CA3) regions are shown in Figure 1e. We also found that SIK1 levels in the DG (*t* = 3.633, *p* = .0070) and CA3 (*t* = 3.004, *p* = .0326) regions were significantly higher in the PTZ‐1 h group than that in the NS group. SIK1 levels in the DG (*t* = 3.383, *p* = .0132) were also significantly higher in the PTZ‐2 h group than that in the NS group (Figure 1f,g). These findings demonstrate that SIK1 was selectively upregulated in the hippocampus after PTZ‐induced seizures.

### 3.2 VPA prevents PTZ‐induced SIK1 increase in the hippocampus of mice

**FIGURE 2:**
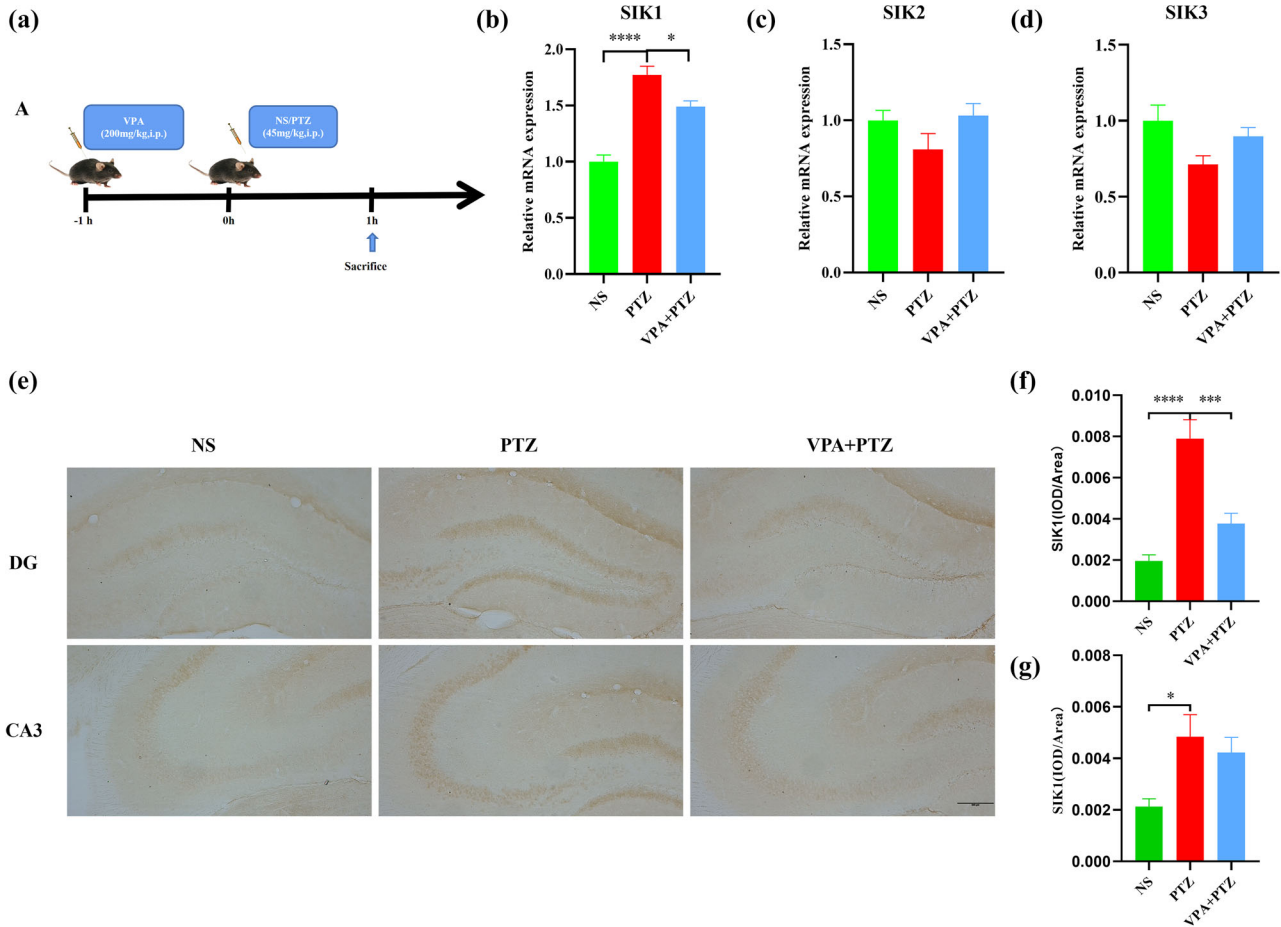
Valproic acid (VPA) prevents salt‐inducible kinase (SIK1) upregulation in the hippocampus of mice with epilepsy (a) pentylenetetrazole (PTZ)‐induced epilepsy treatment options and sampling. VPA injection: mice were intraperitoneally injected with VPA, 200 mg/kg. (b–d) Representative mRNA expression levels of SIK1, SIK2, and SIK3, *N* = 6 per group. (e) Representative immunohistochemistry images of SIK1 in dentate gyrus (DG) and cornu ammonis (CA3) (Bar = 200 µm). (f and g) Quantification of the average optical density (OD) of SIK1 in the DG and CA3 regions of the hippocampus for each group, *N* = 3 per group. ^*^ *p* < .05, ^**^ *p* < .01, ^***^ *p* < .001, ^****^ *p* < .0001.

To explore potential relationship between the expression of hippocampal SIK1 and seizure behavior, we detected the expression of SIKs in the hippocampus of adult epileptic model mice treated with VPA using real time‐PCR (Figures 2b–d). In line with our results, hippocampal SIK1 was increased in the PTZ group (*t* = 8.714, *p* < .0001) compared to the NS group and was remarkably decreased in the VPA + PTZ group (*t* = 3.207, *p* = .019) (Figure 2b). This result was further validated by immunohistochemistry assay, as the SIK1 levels in the DG (*t* = 5.988, *p* < .0001) and CA3 (*t* = 2.723, *p* = .0405) were significantly increased in the PTZ group compared to the NS group and decreased in the DG in VPA + PTZ group compared to the PTZ group (*t* = 4.802, *p* = .0003) (Figure 2e–g). These data suggest that the expression of SIK1 in the hippocampus was positively related to the seizure behavior.

### 3.3 YKL‐06‐061 treatment improves seizure behavior in epilepsy mice

**FIGURE 3:**
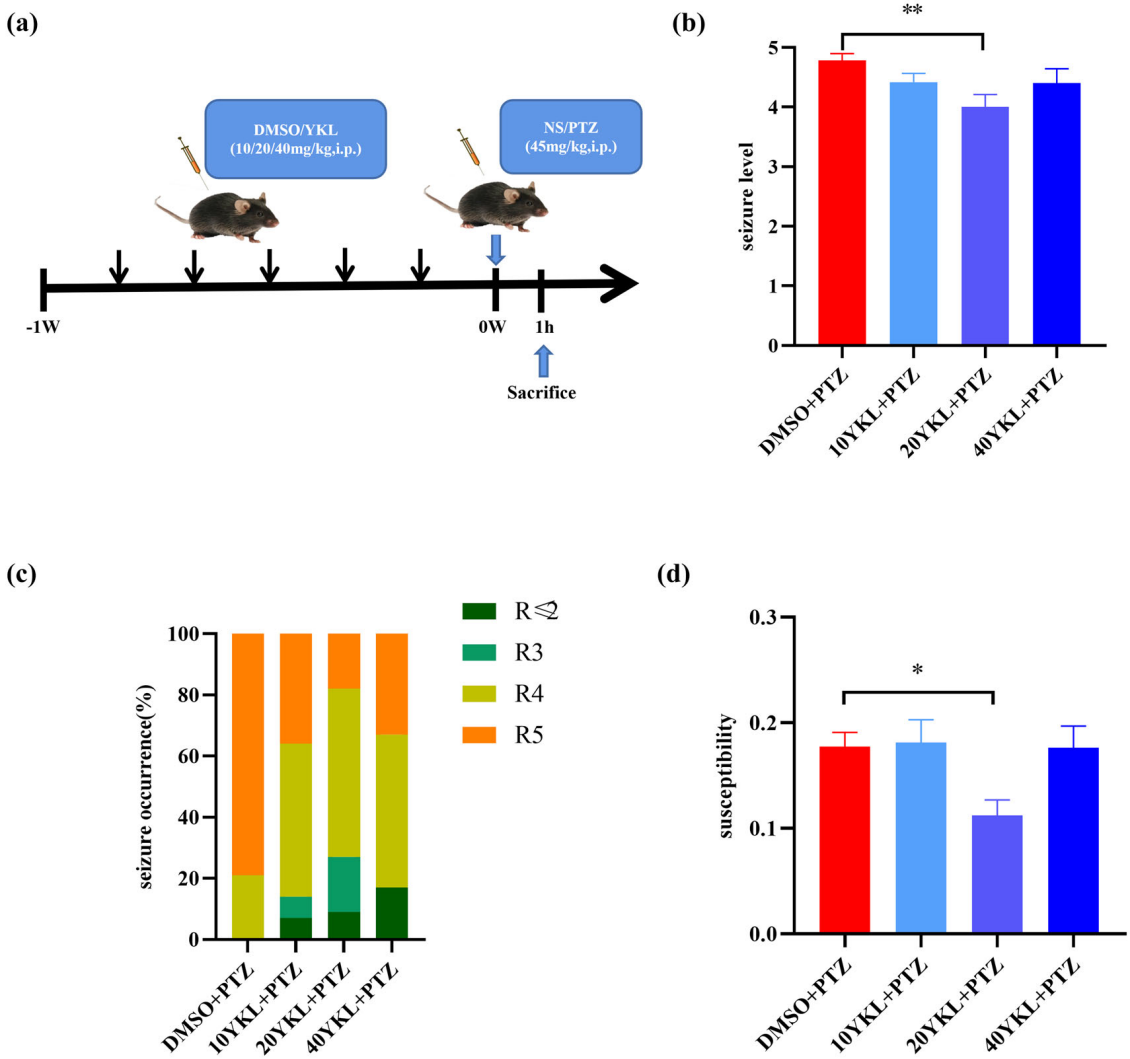
Treatment with different doses of YKL‐06‐061 improved epileptiform behavior in mice with epilepsy: (a) pentylenetetrazole (PTZ)‐induced epilepsy treatment options and sampling; (b) seizure levels in each group; (c) the percentage of seizures in each group; (d) the susceptibility of each group; dimethyl sulfoxide (DMSO) + PTZ (*N* = 14); 10 mg/kg, YKL + PTZ (*N* = 16); 20 mg/kg, YKL + PTZ (*N* = 12); 40 mg/kg, YKL + PTZ (*N* = 10). ^*^ *p* < .05, ^**^ *p* < .01, ^***^ *p* < .001, ^****^ *p* < .0001.

Having demonstrated a possible relationship between SIK1 and seizure behavior, we further investigated whether SIK inhibitors (YKL‐06‐061) could improve the animals’ behavior (Figure 3a). We found that the seizure level was decreased in the 20 mg/kg YKL + PTZ group (*t* = 3.567, *p* = .0061) compared to the DMSO + PTZ group; the percentage of R5 in the 20 mg/kg YKL + PTZ group was significantly reduced, and the percentages of R2, R3, and R4 were increased compared to the DMSO + PTZ group (Figure 3b,c). Moreover, susceptibility was decreased in the 20 mg/kg YKL + PTZ group (*t* = 2.888, *p* = .0444) compared to the DMSO + PTZ group (Figure 3d). These results indicate that blocking SIKs with YKL‐06‐061 could improve the seizure behavior of mice induced by PTZ injection.

### 3.4 YKL‐06‐061 treatment prevents PTZ‐induced activation of IEGs in the hippocampus of mice

**FIGURE 4:**
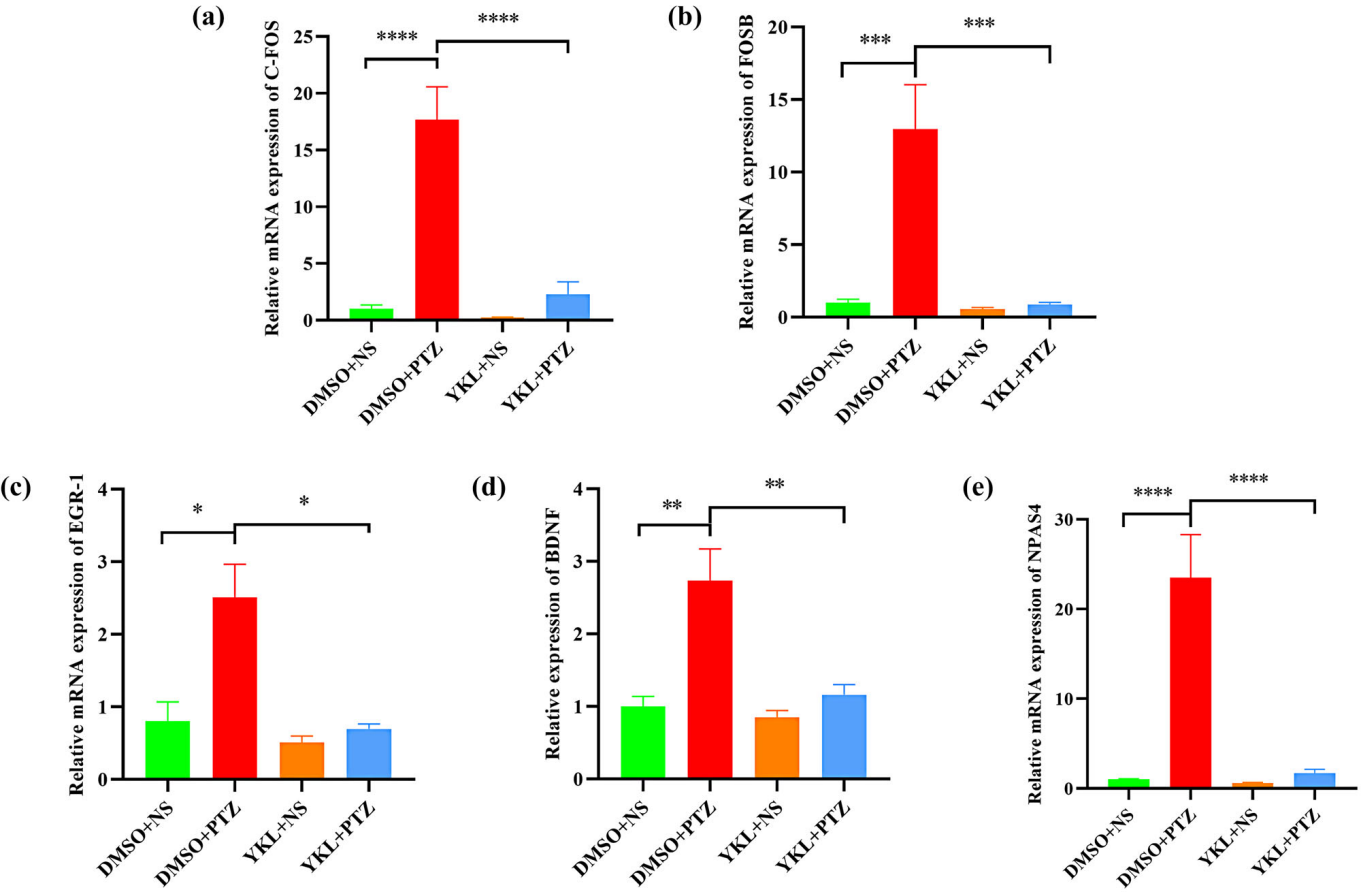
YKL‐06‐061 prevents pentylenetetrazole (PTZ)‐induced activation of immediate early genes (IEGs) in the hippocampus of mice. (a–e) Representative mRNA expression levels of c‐fos, Fos‐B, Egr‐1, brain‐derived neurotrophic factor (BDNF), and neuronal PAS domain protein 4 (NPAS4), dimethyl sulfoxide (DMSO) + normal saline (NS) (*N* = 4), DMSO + PTZ (*N* = 6), YKL + NS (*N* = 4), YKL + PTZ (*N* = 6). ^*^ *p* < .05, ^**^ *p* < .01, ^***^ *p* < .001, ^****^ *p* < .0001.

**FIGURE 5:**
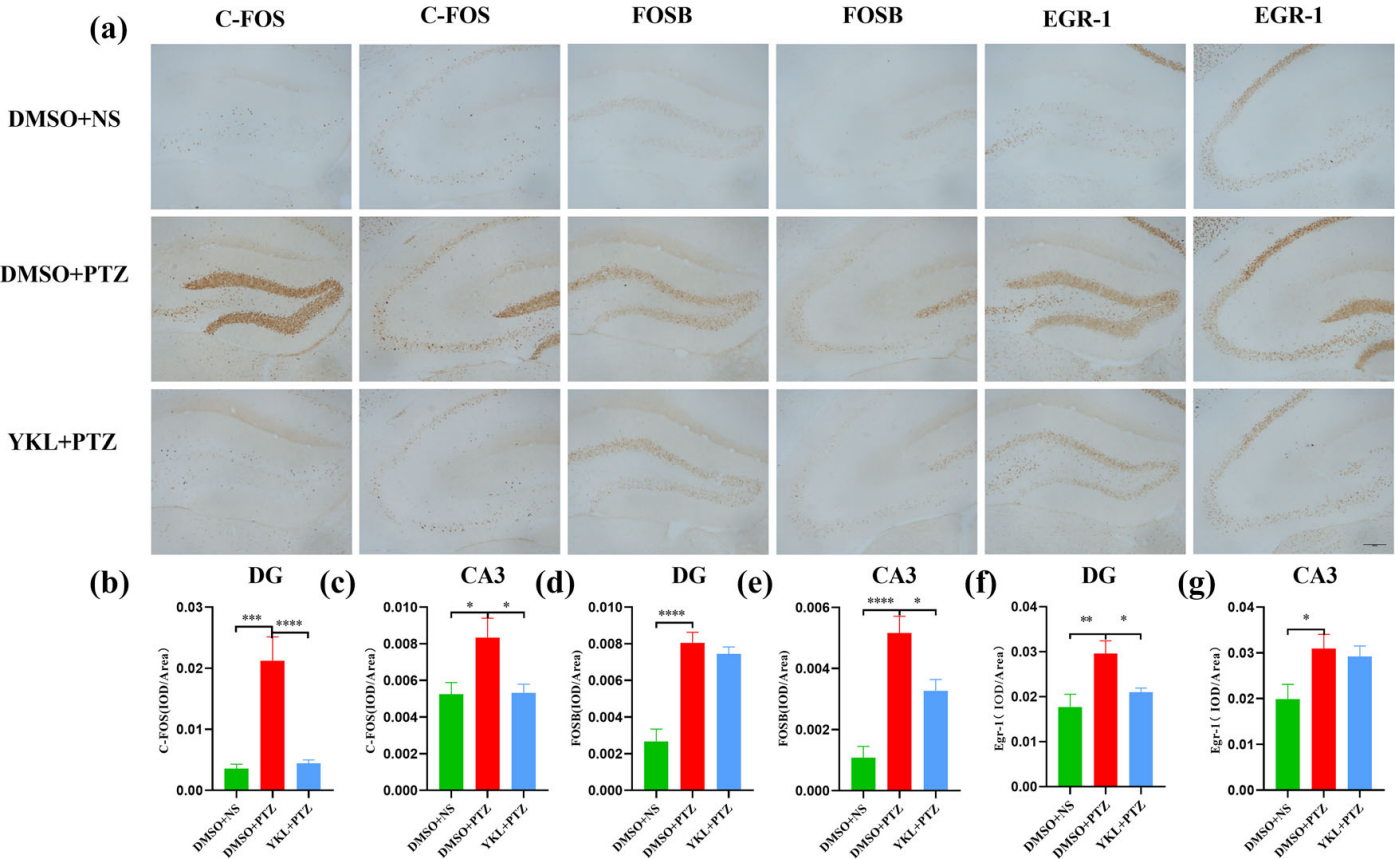
YKL‐06‐061 prevents pentylenetetrazole (PTZ)‐induced activation of immediate early genes (IEGs) in the hippocampus of mice: (a) representative immunohistochemistry images of c‐fos, Fos‐B, and Egr‐1 in the dentate gyrus (DG) and cornu ammonis (CA3) (Bar = 200 µm); (b–g) quantification of the average optical density (OD) of c‐fos, Fos‐B, and Egr‐1 in the DG and CA3 regions of the hippocampus for each group, *N* = 3 per group. ^*^ *p* < .05, ^**^ *p* < .01, ^***^ *p* < .001, ^****^ *p* < .0001.

Real‐time PCR was used to detect the hippocampal mRNA levels of IEGs that were closely related to neuronal overactivation induced by PTZ. As shown in Figure 4a–e, the c‐fos levels (*t* = 7.467, *p* < .0001), Fos‐B levels (*t* = 5.744, *p* = .0004), Egr‐1 levels (*t* = 3.085, *p* = .0453), brain‐derived neurotrophic factor (BDNF) levels (*t* = 4.551, *p* = .0027), and neuronal PAS domain protein 4 (NPAS4) levels (*t* = 6.874, *p* < .0001) were significantly increased in the DMSO + PTZ group compared to the DMSO + NS group, which was reversed by treatment with 20 mg/kg YKL (c‐fos: *t* = 7.547, *p* < .0001; Fos‐B: *t* = 6.114, *p* = .0002; Egr‐1: *t* = 3.798, *p* = .0105; BDNF: *t* = 4.377, *p* = .0038;NPAS4: *t* = 7.020, *p* < .0001). This result was also verified by immunohistochemistry assay, because the integrated optical densities of c‐fos (*t* = 4.245, *p* = .0003), Fos‐B (*t* = 6.837, *p* < .0001), and Egr‐1 (*t* = 3.566, *p* = .0027) were significantly increased in the DMSO + PTZ group compared to the DMSO + NS group, which was reversed by treating DG with 20 mg/kg YKL (c‐fos: *t* = 4.780, *p* < .0001; Egr‐1: *t* = 2.582, *p* = .0397). Meanwhile, the c‐fos levels (*t* = 2.527, *p* = .0481), Fos‐B levels (*t* = 5.642, *p* < .0001), and Egr‐1 levels (*t* = 2.671, *p* = .0315) were significantly increased in the DMSO + PTZ group compared to that in the DMSO + NS group, which was reversed by treating CA3 with 20 mg/kg YKL (c‐fos: *t* = 2.845, *p* = .0219; Fos‐B: *t* = 3.115, *p* = .0112) (Figure 5b–g). These results suggest that YKL‐06‐061 could inhibit neuronal overactivation in the hippocampus of mice following PTZ injection.

### 3.5 YKL‐06‐061 treatment prevents PTZ‐induced activation of IEGs in the prefrontal cortex of mice

**FIGURE 6:**
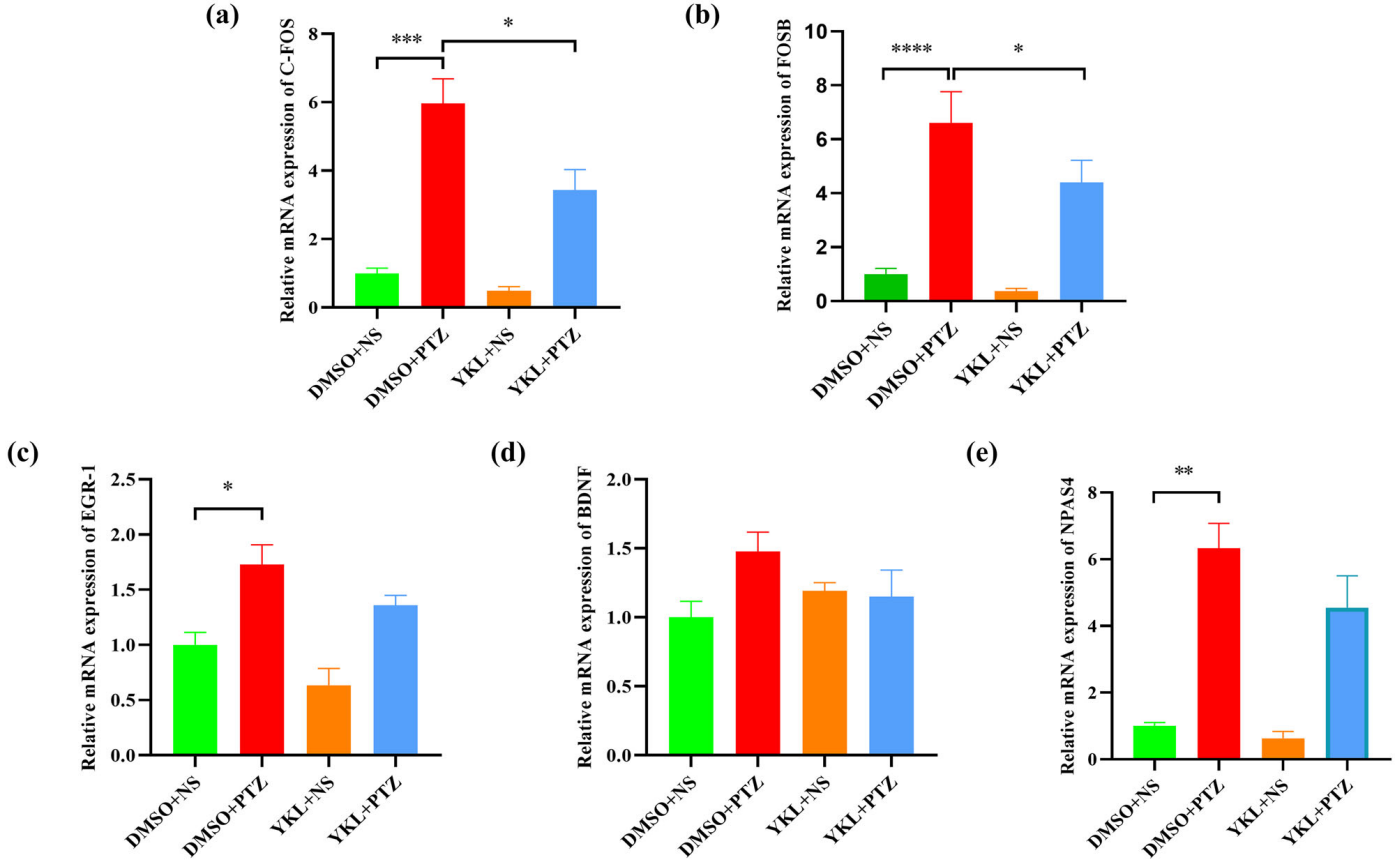
YKL‐06‐061 treatment prevents the activation of immediate early genes (IEGs) in the prefrontal cortex of epileptic mice. (a–e) Representative mRNA expression levels of c‐fos, Fos‐B, Egr‐1, brain‐derived neurotrophic factor (BDNF), and neuronal PAS domain protein 4 (NPAS4), dimethyl sulfoxide (DMSO) + normal saline (NS) (*N* = 4), DMSO + pentylenetetrazole (PTZ) (*N* = 6), YKL + NS (*N* = 4), YKL + PTZ (*N* = 6). ^*^ *p* < .05, ^**^ *p* < .01, ^***^ *p* < .001, ^****^ *p* < .0001.

We also determined the distribution of IEGs in the prefrontal cortex of adult mice using real‐time PCR. As shown in Figure 6a–e, the expressions of c‐fos (*t* = 5.929, *p* = .0001), Fos‐B (*t* = 7.588, *p* < .0001), Egr‐1 (*t* = 3.561, *p* = .0156), and NPAS4 (*t* = 4.920, *p* = .0011) were significantly increased in the DMSO + PTZ group compared to the DMSO + NS group, which was reversed by treating with 20 mg/kg YKL (c‐fos: *t* = 3.376, *p* = .0231; Fos‐B: *t* = 3.348, *p* = .0245).

## 4 DISCUSSION

In the present study, we found that hippocampal SIK levels, specifically those of SIK1, were selectively upregulated in the hippocampus in response to seizures induced by convulsive doses of PTZ. Furthermore, we observed a positive correlation between SIK1 levels and seizure behavior. In addition, mice treated with SIK inhibitors were less prone to seizure onset and showed reduced neuronal activation. Collectively, our data indicate that SIK1 is a critical factor involved in seizure genesis by modulating neuronal excitabilities, thereby providing a potential therapeutic target for epilepsy.

SIKs, including SIK1, SIK2, and SIK3, belong to the AMPK family (Badawi et al., 2021). However, compared to other well‐established energy‐response kinase AMPK, the roles of SIKs in human diseases, especially epilepsy, have rarely been investigated. Because the expression of SIKs has not been systematically studied in the epileptogenic brain, we first studied their mRNA and protein levels in the hippocampus of mice challenged with PTZ, which is clinically relevant to human temporal lobe epilepsy and generalized tonic–clonic convulsions (Kumar & Kumar, 2017). After confirming that PTZ (45 mg/kg) (Shan et al., 2018) induced seizures characterized by tonic convulsions, jumping, and/or running in our mouse model, we observed the expression of SIKs 1, 2, and 3 at different time points post‐PTZ injection. Interestingly, only SIK1 was significantly enhanced after PTZ injection, which was validated using immunohistochemical assays. In fact, the expression of SIK1, but not SIK2 or SIK3, is activity‐dependent because KCl treatment induced the upregulation of SIK1, but not SIK2 or SIK3 in cultured neurons (Li et al., 2009). Moreover, 1 h of restraint stress increased SIK1 mRNA levels, whereas SIK2 mRNA levels showed only minor increases (Liu et al., 2012). Following KA‐induced seizures, the levels of SIK1 mRNA increased throughout the hippocampal formation as early as 1 h after seizure onset (Feldman et al., 2000), which was corroborated by our data. We further used VPA, a currently available antiepileptic drug (Loscher, 2002), to explore whether the expression of SIK1 was positively related to seizure behavior. VPA is the first‐choice drug for the treatment of epilepsy because it has the broadest spectrum of anticonvulsant action in both adults and children. In addition, VPA counteracts the neuronal damage induced by prolonged seizures during hippocampal formation (Romoli et al., 2019). Consistent with the results of a previous study, VPA effectively prevented seizures in PTZ‐treated mice. Importantly, we found that the expression of SIK1 was also downregulated in VPA‐treated mice; however, whether other anticonvulsant drugs alter the expression of SIK1 needs further investigation. Taken together, these results suggest that changes in the expression of SIK1 after an insult may contribute to epileptogenesis.

However, whether normalizing SIKs is a therapeutic measure for epilepsy has not yet been reported. This prompted us to compare the anti‐seizure efficacy of inhibitors of SIK. In this study, we used the recently developed inhibitor YKL‐06‐061, which has a high affinity for SIK1 and 3, but to a lesser extent for SIK2, and tested its effects on seizure development in rodent epilepsy models. We found that YKL‐06‐061 exerts an antiepileptic effect on PTZ‐induced epileptiform seizures. However, we also noted that mice treated with 40 mg/kg YKL‐06‐061 showed enhanced mortality, which might be a negative feedback signal, because SIK1 induction could also prevent persistent transcription during long‐lasting neuronal activity (Li et al., 2009). We consider that the increase in susceptibility score again at 40 mg/kg may be related to its high mortality rate; however, the specific reasons still require further exploration. Since there is little information regarding the role of YKL‐06‐061 in brain function, we could only conclude that the optimal dose of YKL‐06‐061 for the control of seizures may be 20 mg/kg. However, whether a double or higher dose leads to other toxic effects requires systematic evaluation. In addition, we cannot exclude the role of SIK2 and SIK3 in the development of seizure behavior. Boosting the glial SIK3 K^+^ buffering pathway suppresses seizures (Lones & DiAntonio, 2023) and YKL‐06‐061 is a pan‐inhibitor of SIK1, SIK2, and SIK3 (Mujahid et al., 2017); a more selective SIK1 inhibitor or knockdown of SIK1 needs to be evaluated in detail in future studies.

Hippocampal and cortical hyperexcitability are the defining features of most epilepsies (Zhang et al., 2010), but the molecular mechanisms regulating excitability in these brain regions are not fully understood. Excessive neuronal stimulation induced by epileptic seizures results in rapid and dramatic changes in the expression of IEGs (Kalinina et al., 2022), which can mediate direct effects on neuronal excitability and function. Inhibition of IEG induction by antisense oligonucleotides can disrupt seizure development in several animal models of epilepsy (Chiasson et al., 1998; Panegyres & Hughes, 1997; Rocha & Kaufman, 1998; Rocha et al., 1999; Suzukawa et al., 2003). The expression of c‐fos, Egr‐1, and Fos‐B is an indirect marker of neuronal activity, which often occurs when neurons generate action potentials (Lin et al., 2018; You et al., 2017). Our results showed enhanced expression of hippocampal IEGs (including c‐fos, Fos‐B, and Egr‐1) in PTZ‐treated mice, consistent with the findings of previous studies (Chiasson et al., 1998; Panegyres & Hughes, 1997; Rocha & Kaufman, 1998; Rocha et al., 1999; Suzukawa et al., 2003). Notably, pretreatment with 20 mg/kg YKL‐06‐061 significantly reduced the expression of hippocampal IEGs in mice challenged with PTZ.

Our study has several limitations. Further work is needed to delineate the specific downstream mechanisms of SIK1 activation that would induce neuronal hyperactivity. Inhibitors of SIKs should be systematically tested against classical models such as maximal electroshock, acquired epilepsies such as traumatic brain injury‐induced seizures, as well as in models of treatment‐resistant seizures, such as Dravet mice (Kalume et al., 2013). We also realize that the lack of electroencephalogram (EEG) recordings is a significant limitation of the present study, because EEG recordings might provide more information (such as onset of electrographic seizures and percentage of electrographic seizure duration) regarding the alteration of the epileptic mice model. Although glial cell‐induced inflammation is also involved in the development of epilepsy (Henning et al., 2023; Vezzani et al., 2022), the present study primarily focused on the role of SIK1 in the regulation of neuronal overactivation. Finally, whether inhibitors of SIKs would continue to function after the onset of epilepsy or in a chronic seizure model requires further study.

Acknowledging the limitations noted above, our findings provide the first evidence that SIK1 affects gene regulation in neuronal hyperactivity, which is involved in seizure behavior. Targeting SIK1 through the development of selective inhibitors may lead to disease‐modifying therapies that reduce epilepsy progression.

## AUTHOR CONTRIBUTIONS

Lixuan Peng and Yang Xu performed PCR tests, Wenyu Cao and Xiaohan Tang performed immunohistochemistry tests, and Lixuan Peng, Yuyan Xiang, and Wenyu Cao established the animal model and administered YKL‐06‐061 intervention. Lixuan Peng and Cai Li performed statistical analyses. Lixuan Peng and Wenyu Cao drafted the manuscript. Lixuan Peng, Huamao Zhou, Wenyu Cao, and Suyun Li designed the study and revised the manuscript. All authors read and approved the final manuscript.

## CONFLICT OF INTEREST STATEMENT

The authors declare no conflicts of interest.

### PEER REVIEW

The peer review history for this article is available at https://publons.com/publon/10.1002/brb3.3305.

## Data Availability

The datasets used and/or analyzed in the current study are available from the corresponding author upon reasonable request.
